# Ethical acceptability of offering financial incentives for taking antipsychotic depot medication: patients’ and clinicians’ perspectives after a 12-month randomized controlled trial

**DOI:** 10.1186/s12888-017-1485-x

**Published:** 2017-08-29

**Authors:** Ernst L. Noordraven, Maartje H. N. Schermer, Peter Blanken, Cornelis L. Mulder, André I. Wierdsma

**Affiliations:** 1 Dual Diagnosis Center (CDP) Palier, Parnassia Psychiatric Institute, 2552 KS The Hague, The Netherlands; 2 Department of Psychiatry, Erasmus University Medical Center, Epidemiological and Social Psychiatric Research Institute, 3015CE Rotterdam, The Netherlands; 3 Bavo-Europoort Mental Health Care, 3066 TA Rotterdam, The Netherlands; 4 Parnassia Addiction Research Centre (PARC), Brijder Addiction Treatment, Parnassia Psychiatric Institute, 2553 RJ The Hague, The Netherlands

**Keywords:** Schizophrenia, Ethics, Financial incentives, Antipsychotic depot medication

## Abstract

**Background:**

A randomized controlled trial ‘Money for Medication’(M4M) was conducted in which patients were offered financial incentives for taking antipsychotic depot medication. This study assessed the attitudes and ethical considerations of patients and clinicians who participated in this trial.

**Methods:**

Three mental healthcare institutions in secondary psychiatric care in the Netherlands participated in this study. Patients (*n* = 169), 18–65 years, diagnosed with schizophrenia, schizoaffective disorder or another psychotic disorder who had been prescribed antipsychotic depot medication, were randomly assigned to receive 12 months of either treatment as usual plus a financial reward for each depot of medication received (intervention group) or treatment as usual alone (control group). Structured questionnaires were administered after the 12-month intervention period. Data were available for 133 patients (69 control and 64 intervention) and for 97 clinicians.

**Results:**

Patients (88%) and clinicians (81%) indicated that financial incentives were a good approach to improve medication adherence. Ethical concerns were categorized according to the four-principles approach (autonomy, beneficence, non-maleficence, and justice). Patients and clinicians alike mentioned various advantages of M4M in clinical practice, such as increased medication adherence and improved illness insight; but also disadvantages such as reduced intrinsic motivation, loss of autonomy and feelings of dependence.

**Conclusions:**

Overall, patients evaluated financial incentives as an effective method of improving medication adherence and were willing to accept this reward during clinical treatment. Clinicians were also positive about the use of this intervention in daily practice. Ethical concerns are discussed in terms of patient autonomy, beneficence, non-maleficence and justice. We conclude that this intervention is ethically acceptable under certain conditions, and that further research is necessary to clarify issues of benefit, motivation and the preferred size and duration of the incentive.

**Trial registration:**

Nederlands Trial Register, number NTR2350.

**Electronic supplementary material:**

The online version of this article (10.1186/s12888-017-1485-x) contains supplementary material, which is available to authorized users.

## Background

Patients with psychotic disorders often have problems adhering to their prescribed antipsychotic medication [1], making it difficult to control their psychiatric symptoms. Non-adherence has also been associated with an increased risk of hospital admissions, suicide attempts, violence, self-harm, substance use and treatment costs [2–4]. Unfortunately, interventions to improve adherence such as psychoeducation or adherence therapy have not been consistently successful for patients with schizophrenia [5].

Although providing financial incentives for taking antipsychotic depot medication is an effective intervention for improving adherence [6–9], clinicians and healthcare workers have several ethical reasons for criticising the use of financial incentives among patients with severe mental illnesses. For example, 76% managers of assertive outreach teams surveyed in England stated one or more ethical reason for refusing to provide this intervention [10]. First, patients may feel bribed or coerced into taking their medication – because they need income, for example [11]. Second, the therapeutic relationship might be damaged if patients receive financial incentives, possibly undermining voluntary medication adherence [6]. Finally, if illness or medication impairs patients’ decision-making capacities, it is unreasonable to expect them to make informed decisions about their treatment process, including the incentive [12]. This raises ethical concerns about respecting patient autonomy.

Offering financial incentives may also have negative consequences on patients’ intrinsic motivation for treatment [13–15]. If financial incentives are offered only to non-adherent patients and are eventually removed, patients could refuse all medication (‘crowding out effect’) [16] or may deliberately become non-adherent in order to receive financial incentives again [17]. Similarly, many patients with severe mental illnesses are vulnerable or have impaired decision-making capacity, which sometimes gives clinicians the feeling they are ‘buying’ their patients.

A focus group study by Priebe and colleagues [18] explored the attitudes of different stakeholders (i.e. patients, psychiatrists, nurses, social workers, psychologists and multidisciplinary teams) towards the ethical acceptability of financial incentives. They identified several themes – including coercion, effectiveness and perverse incentive – that dominated the discussion. Each stakeholder group tended to indicate the same discussion threads. However, few patients (*n* = 27) participated in this study, and neither patients nor clinicians had any actual experience of using financial incentives.

As prior research has thus focused on clinicians’ beliefs about applying this intervention [19], greater attention should be paid to evaluating the ethical concerns and considerations of patients who have had practical experience of financial incentives. We therefore report on patients and clinicians who participated in *Money for Medication*, a randomized controlled trial [9] in which patients were offered financial incentives for taking antipsychotic depot medication for a 12-month intervention period. The opinions of patients and clinicians on this intervention were organized on the basis of the four-principles approach of Beauchamp and Childress [20]. This pragmatic approach [21] was used to categorize ethical arguments into one of the following ethical principles: autonomy (the right of competent patients to make their own decisions, and the obligation to respect the decision-making capacities of autonomous persons); beneficence (the obligation to provide benefits and to balance benefits against risks); non-maleficence (the obligation to avoid causing harm); and justice (the obligation of fairness in the distribution of benefits and risks).

## Methods

### Design

Data were collected in the context of *Money for Medication* (M4M), a multicentre, open-label, parallel-group, randomized controlled trial [9]. The study was approved by the accredited Dutch Medical Ethical Trial Committee at Erasmus University Medical Center (NL31406.097.10; file number P13.258) and registered with the Netherlands Trial Register (NTR2350).

### Participants

Participants were included at three mental health-care institutions in secondary psychiatric care services in the Netherlands: the Dual Diagnosis Center (CDP) Palier, Parnassia, and BavoEuropoort. Primarily these organisations treat patients with psychotic and other severe mental disorders (often with comorbid substance use). In general, at the start of the study, these patients received voluntary treatment and are motivated by the clinicians to accept their medication. Involuntary outpatient treatment was not an in- or exclusion criterion for participation in the study. Eligible patients were aged between 18 and 65 years, had a psychotic disorder (such as schizophrenia, schizoaffective disorder or another psychotic disorder) classified by psychiatrists using the DSM-IV; had been prescribed antipsychotic depot medication or had an indication to start using depot medication; and were participating in outpatient treatment. All patients had given written informed consent before the baseline interview was conducted. Exclusion criteria were: inability to participate due to cognitive impairments (as determined by the clinicians on the basis of their clinical judgement or stated in the patients’ record) and/or insufficient understanding of Dutch (observation of research assistants during interviews). Patients who initially met the inclusion criteria were informed about the study by their clinicians and were asked to participate. If a patient declined to participate, this decision was registered anonymously to allow assessment of selection bias.

### Procedure and data collection

In total, 879 patients from the mental healthcare teams were assessed for eligibility; 710 patients were excluded because:(1) they had no prescription or indication for antipsychotic depot medication (*n* = 460), (2) did not react to requests for participation in the trial (*n* = 101), (3) refused participation (*n* = 28), (4) or various other reasons (*n* = 121), including being admitted to a hospital, moving house to a different city, transfer to another treatment team, imprisonment, or insufficient information to be contacted. Between May 21, 2010 and October 15, 2014, 169 patients were randomly allocated to 12 months of experimental treatment (M4M) or to 12 months of treatment as usual (TAU; control condition). Randomisation was stratified by treatment site and suspected prognostic factors: sex, comorbid substance-use disorder (absent vs present), and compliance with antipsychotic medication in the 4 months before baseline (<50% vs ≥50%). During the intervention period, no patients refused participation due to the content of the study. TAU consisted of outpatient treatment provided by community mental-health teams and flexible assertive-community-treatment teams.

Patients in the intervention group (M4M) received treatment as usual, plus a financial reward each time they received their prescribed depot of antipsychotic medication during the 12-month experimental study phase. The maximum amount they received was €30 per month. The rewards were paid out by the patients’ treating nurses as soon as the patient had received the injection or had swallowed the penfluridol. The methods and results of the study have been described in Noordraven et al. [9].

### Assessment and questionnaire

After the 12-month intervention period, patients’ and clinicians’ attitudes and opinions were assessed using a short questionnaire constructed for the study. The first question asked whether this intervention was a ‘good idea’ or a ‘bad idea’. Next, two open-ended questions asked about the advantages and disadvantages of using financial incentives. Nineteen statements then addressed ethical considerations on the consequences of M4M on topics such as the therapeutic relationship, intrinsic motivation, inequality between patients, and patient vulnerability (Additional file 1 shows our complete questionnaire). Items were scored on a 5-point scale (1 = strongly disagree, 5 = strongly agree) and dichotomized into ‘disagree’ (scores 1, 2, 3) and ‘agree’ (scores 4 and 5). To assess the effect of alternative breakpoints, sensitivity analyses were performed; no relevant differences were found. All interviews were conducted by extensively trained research assistants (Master’s-level psychologists).

To explore the different components of ethical concerns, each statement was categorized post-hoc into one of the four ethical principles (Table 1): ‘autonomy’ (items 4, 7, 18; expressed in statements such as ‘*if they receive money for their depot medication, patients will feel forced to accept their depots’* and *‘it is not permissible to buy patients by giving them money to take their medication’*); ‘beneficence’ (items 1, 2, 3, 5, 6, 11, 15, 19; expressed in statements such as ‘*patients will accept their depots more often if they receive money*’ and ‘*money for depots improves patients’ motivation to use depot medication*’); ‘non-maleficence’ (items 9, 12, 13, 14; expressed in statements such as ‘*Money for depots is harmful to the therapeutic relationship*’ and ‘*if someone receives money for his depot, he won’t gain insight into his problems*’); and ‘justice’ (item 8, expressed in a single statement: ‘*jealousy will arise if some patients receive money for their depots and others do not*’). Statements that did not fit the description of these principles were labelled as ‘other considerations’ (items 16, 17).

**Table 1:** Patients’ and clinicians’ agreement with ethical aspects of the Money for Medication intervention to improve medication adherence

Ethical concern	Statement	Patients (*N* = 133)	Clinicians (*N* = 97)
	*‘Patients will feel dependent if they receive money for their depots’*	33% (44)	31% (30)
Autonomy	*‘If they receive money for their depot medication, patients will feel forced to accept their depots’*	36% (47)	27% (26)
	*‘It is not permissible to buy patients by giving them money to take their medication’*	21% (27)	16% (15)
Beneficence	*‘To give money for depots is good’* *‘Giving money for depots emphasizes the things that are going well’* *‘Money could just be the right push to accept your depot’* *‘Money for depots improves patients’ motivation to use depot medication’* *‘Money for depots will work in daily practice’* *‘Money for depots helps to get into a positive flow’* *‘Patients will accept their depots more often when they receive money’* *‘Money for depots is beneficial for patients wellbeing’* *‘If patients receive money for their depot they will accept it more often’*	68% (91) 51% (66) 72% (96) 72% (95) 67% (89) 62% (81) 79% (103) 58% (76) 75% (98)	47% (46) 33% (32) 88% (85) 82% (80) 53% (51) 61% (59) 72% (70) 42% (41) 79% (77)
	*‘If someone receives money for his depot, he won’t gain insight into his problems’*	23% (30)	35% (34)
	*‘Money for depots is harmful to the therapeutic relationship’*	16% (21)	16% (16)
Non-maleficence	*‘If patients no longer receive money for their depots they will stop to accept their depots’* *‘Money for depots will provoke patients to follow their treatment less for themselves but more for the money’*	36% (47) 38% (49)	37% (45) 71% (69)
Justice	*‘Jealousy will arise if some patients receive money for their depots and others do not’*	62% (82)	71% (69)
Other	*‘Giving money for depots is ethically acceptable’*	49% (64)	34% (33)
	*‘It is good to reward good behavior with money’*	76% (99)	38% (37)

We also described the commonest advantages and disadvantages named spontaneously by patients and clinicians in response to the open-ended questions. Using descriptive statistics, we identified differences between patients and clinicians, and explored the differences between patients who had actually received the intervention (M4M group) and those who had not (TAU group). All analyses were performed using SPSS version 21.0.

## Results

After the 12-month intervention period, interview data were available for 133 patients and 97 clinicians (Table 2). Patients lost to follow-up showed no significant differences relative to patients who had outcome data after 12 months, and were distributed equally across conditions (17 intervention and 19 control). Because of (repeated) non-attendance, we were unable to conduct follow-up interviews for these patients. However, they did not actively withdrew their consent to participate in our study, nor did they report any (ethical) concerns as reason for non-attendance. Overall, 88% of the patients and 81% of the clinicians reported that the M4M project was a good idea. These percentages were similar between patients from the intervention group (92%) and the control group (84%).

**Table 2:** Characteristics of patients and clinicians

Variable	Patients (*n* = 133)	Clinicians (*n* = 97)
Age, mean (SD) years	40.3 (9.4)	41.5 (12.5)
Gender, *N* (%)		
Male	99 (74)	35 (37)
Duration of illness mean (SD), years	12.1 (8.3)	-
Diagnosis, *N* (%)		
- Schizophrenia paranoid type	75 (56.4)	-
- Schizoaffective disorder	15 (11.3)	-
- Psychotic disorder NOS	14 (10.5)	-
- Schizophrenia disorganized type	9 (6.8)	-
- Other schizophrenic disorder	20 (15.0)	-
Working experience, mean (SD), years	-	14.4 (11.4)
Job description, *N* (%)		
- Psychiatrist	-	9 (9)
- Psychologist	-	11 (11)
- Social worker	-	15 (15)
- Social psychiatric nurse	-	20 (21)
- Nurse	-	25 (26)
- Intern	-	9 (9)
- Other	-	8 (8)

### Patients versus clinicians

#### Autonomy

Around 33% of the patients and clinicians reported that if patients were offered money, they would feel dependent, pressured or coerced to accept their antipsychotic depots.

#### Beneficence

Patients (79%) and clinicians (72%) believed that patients would be more adherent to their antipsychotic depot medication if financial incentives were used. Similarly, a majority of patients (72%) and clinicians (82%) agreed that ‘money for depots would improve the motivation to use depot medication’. However, only 58% of patients and 42% of clinicians agreed with the statement that ‘money for depots is beneficial for patients wellbeing’.

#### Non-maleficence

Although few patients (23%) agreed with the idea that ‘if someone receives money for his depot, he won’t gain insight into his problems’, more clinicians (35%) were worried about this negative consequence. While clinicians (71%) also agreed with the statement that financial incentives would provoke patients into follow treatment less for themselves but more for the money, this opinion was shared by fewer patients (38%). A majority of the patients (84%) and clinicians (84%) stated that they did not expect the therapeutic relationship to suffer from the use of monetary rewards.

#### Justice

This statement referred to the obligation to treat like cases alike. A majority of the patients (62%) and clinicians (71%) believed that jealousy would occur if some patients received money for their depots but others did not.

#### Other considerations

While most patients (76%) agreed with the statement that it would be good to reward good behaviour with money, fewer clinicians (38%) did so. Nearly half the patients (49%) agreed that giving money for depots was ethically acceptable, an opinion shared by a third of clinicians (34%).

#### Between patients: Experimental group versus control group

Overall, the ratings on the questionnaire items did not differ significantly between intervention and control patients. Intervention patients rated M4M somewhat more positively than control patients on only two items: ‘money for depots will work in daily practice’ (77% vs. 59%) and it is ‘ethically acceptable to give money for depots’ (57% vs. 41%).

### Spontaneously reported advantages and disadvantages

Eighty-two patients and 87 clinicians spontaneously reported five advantages of using financial incentives: *increased compliance* (15 patients (18%) vs. 40 clinicians (46%)); *increased motivation for treatment* (15 patients (18%) vs. 32 clinicians (37%)); *more money to spend* (34 patients (41%) vs. 5 clinicians (6%)); *more time to talk with patients* (11 patients (13%) vs. 1 clinician (1%)); and *improved illness insight* (7 patients (9%) vs. 9 clinicians (10%)). Similarly, 27 patients and 74 clinicians named five potential disadvantages: *becoming dependent and refusing all depots when financial incentives were no longer offered* (7 patients (26%) vs. 24 clinicians (32%)); *becoming externally motivated for treatment* (7 patients (26%) vs. 23 clinicians (31%)); *the unfairness of some patients not receiving financial incentives* (10 patients (37%) vs. 6 clinicians (8%)); *the possibility that patients would not gain illness insight* (1 patient (4%) vs. 12 clinicians (16%)); and *the risk that patients would use the money to buy drugs* (2 patient (7%) vs. 8 clinicians (11%)).

## Discussion

This 12-month randomized controlled trial explored patients’ and clinicians’ ethical concerns regarding the use of financial incentives to improve adherence to antipsychotic depot medication. In general, patients viewed such incentives as an effective method of improving their adherence, and were willing to accept them during clinical treatment. Clinicians were also positive about using this intervention in clinical practice, and had ethical concerns that were similar to those identified by their patients.

However, even though they reported that offering financial incentives was a good idea, only a minority of the clinicians and about half of the patients believed this intervention to be ethically acceptable. These answers show the complex and possible ambivalent attitudes about using financial incentives. Patients might be pragmatic and state that they think of this intervention as a ‘good idea’; believing it will be efficient in daily practice, for example. At the same time, independent from this practical oriented vision, they might believe this intervention is not an ethical practice. Although these results may seem contradictory, they may be two sides of the same coin. An example to illustrate this point is the classic trolley thought-experiment [22]: a dilemma between killing 1 person in order to save 5. Most people would reason from a rational point of view this would be a valid choice to make (‘good idea’), while at the same time, still believing that it is overall unethical to end another persons’ life (‘ethical acceptability’). In this study, patients and clinicians believed that monetary payments would improve medication adherence, even though both groups were worried that jealousy would occur if some patients received monetary payments and others did not. Ethical concerns were grouped on the basis of the four-principles approach in terms of patient autonomy, beneficence, non-maleficence and justice.

### Autonomy

From a consequentialist standpoint, a given intervention would be legitimate if the outcomes were beneficial. In this case, financial incentives increased medication adherence, which was the primary aim of the study [9]. However, we would argue that it is not sufficient to focus only on the outcomes of an intervention: the act of giving financial incentives itself should also be evaluated independently of its consequences. Regardless of the outcomes, ‘bribing’ or coercing patients into using medication would be ethically problematic. To respect patients’ autonomy, one should therefore evaluate the amount of money being offered [23]. If, for example, patients were offered €1000 per depot taken (whether weekly or monthly), they would likely cross their personal boundaries more easily, and might feel coerced into accepting their medication. Particularly among patients with a psychosis, who are often in need of financial support, the payment should not be that high to make them feel forced to accept medication [24].

While some 30% of the patients in this study believed that patients would feel forced or dependent if offered monetary payments, we would argue that it is legitimate to offer incentives that make patients feel slightly pressured –but not coerced or manipulated- to take their depots [25]. We also believe that outright coercion was not involved in our study, since no consequences were attached to rejection of the medication and financial reward: patients who rejected depots were not forced to take their medication, nor were they admitted involuntarily. In practice, the size of the payment should be chosen in a way that always leaves patients with a fair opportunity to say no, if they really do not want to do something. To respect patient autonomy and prevent coercion, the use of incentives should not be considered before carefully weighing the size of the payment in relation to the specific patient population, and before considering whether the incentive being offered is unconditional.

### Beneficence

A majority of the patients and clinicians were convinced that offering financial incentives would increase adherence to antipsychotic depot medication. However, only around 50% of them believed this intervention would also benefit patients’ wellbeing. The fact that the increased medication adherence in this study did not lead to improved clinical outcomes, such as better quality of life or fewer hospital admissions [9], shows a complex association between adherence and patients’ wellbeing. This makes it difficult to conclude whether this intervention is truly beneficial. Offering financial incentives did benefit the number of accepted depots – which was our primary aim – and patients and clinicians alike acknowledged that patients’ motivation to accept their medication had improved.

Although medication non-adherence has been shown to be associated with various negative clinical outcomes, such as increased risk of hospital admissions, suicide attempts, violence or substance abuse, our study did not improve clinical outcomes. We would nonetheless argue in favour of using monetary payments: they are effective for improving medication adherence and there are various reasons why we did not find improvements in clinical outcomes. While these reasons have been discussed in depth elsewhere [9], they should be summarized briefly here. First, our overall study was designed primarily to improve adherence and to study the effectiveness of using financial rewards. At 12 months, the study may have been too short to detect any improvements in clinical symptoms, especially among chronically ill patients with a mean illness duration of about 12 years. But we should also note that clinical outcome measures did not get worse during the intervention period, and there were no indications that M4M negatively affected patient autonomy or therapeutic relationships. Therefore, we believe that it is ethically acceptable to use M4M in clinical practice, even though we found no improvements on clinical outcomes.

### Non-maleficence

An important concern in previous studies is that financial incentives would undermine the therapeutic relationship between patients and clinicians. Clinicians might feel reluctant to offer payments if these could damage or disrupt the relationships with their patients, which are often built with great difficulty and over long periods of time. After the 12-month intervention period, however, patients and clinicians did not report any indications that the therapeutic relationship had suffered from the use of monetary rewards. In addition, patients in the M4M condition did not report more side effects of medication, nor did they use more alcohol or illicit drugs than patients in the control group.

Another ethical concern was the concept of ‘motivation’. Clinicians believed that patients would follow treatment less for themselves and more for the money. They are worried that externally motivated patients will stop to take their medication when incentives are no longer given, since it has been shown that initial positive behaviour changes cannot be sustained after withdrawal of external rewards [26]. Patients, however, disagreed with these concerns. Furthermore, we found that during the 6 month follow-up period, the intervention group still accepted more depots than patients from the control group. This shows that financial incentives can be discontinued without the danger of patients becoming completely non-adherent or less adherent than before receiving financial payments.

### Justice

While some patients received money and other patients did not, a majority of patients and clinicians reported that jealousy could occur. If this intervention were used only with non-adherent patients, this could lead adherent patients to become non-adherent on purpose, or to complain about unequal treatment. Clinicians also suspected that patients might reject their medication if payments were no longer offered, while patients themselves did not expect this to happen. For reasons of justice and to overcome this problem of inequality, we therefore recommend that all patients are offered payments for accepting their depot medication [23] – as in our study – without making distinctions based on previous levels of adherence.

### Strengths and limitations

Our study is one of the first to collect empirical data on patients’ and clinicians’ opinions *after* patients have received financial incentives for accepting antipsychotic depot medication. This is important: the opinions of patients with psychotic disorders are often overlooked, but are crucial if we wish to improve treatment. Another advantage of our study is that patients and clinicians all experienced the intervention in daily practice. Their opinions were thus based in practice much more than if the intervention had merely been discussed hypothetically.

A limitation of the study is that our questionnaire was not constructed on the basis of a previously defined theoretical model: instead, we retrospectively categorized each statement into one of the four main ethical principles and explored patients’ and clinicians’ different views on the intervention. Another limitation is that selection bias may have occurred with respect to the total population of patients on depot medication. Patients who participated might have been biased towards a more positive attitude on using financial incentives, simply because all patients wanted to participate in this study, and showed up for appointments to conduct our interviews. However, we found no differences between patients who actually received financial incentives (M4M group) and those who did not (TAU group).

## Conclusions

In clinical practice, patients and clinicians were positive about the use of financial payments to improve adherence to antipsychotic depot medication. Importantly, the fear that financial incentives would harm the therapeutic relationship was not confirmed. At the same time, however, more than half of the patients and clinicians reported to have ethical concerns (e.g. jealousy or reduced illness insight). Therefore, we consider the use of monetary incentives to take anti-psychotic depot medication to be ethically acceptable on four conditions: the amount offered should be moderate, the offer should be unconditional (i.e. there are no consequences if the patient refuses); the incentives should be made available to all patients; and a monitoring system should be in place to track changes in patients’ health and/or well-being.

However, it still remains unclear to what extent this type of intervention affects internal and external motivation for treatment, and for how long monetary payments should be offered. Our results showed that when financial payments were no longer offered, most patients from the intervention group continued to have improved adherence rates, whereas others relapsed. This indicates that for most patients, temporary incentives might be sufficient to improve their motivation for medication intake over a longer period of time, while for others, continuous payments might be more suitable to maintain higher adherence rates. Longer follow-up periods are needed to examine whether sustained improved adherence might be associated with better clinical outcomes. For practical purposes, however, and to prevent difficulties (e.g. jealousy, inequality between patients, risks of becoming non-adherent on purpose in order to receive incentives), we recommend offering financial incentives to all patients without making distinctions.

Future research should also examine the optimal level of incentives; if incentives are too substantial, this could increase the likelihood of bribing patients into doing something they might not want, instead of offering them an independent choice. Also, higher incentives might harm the therapeutic relationship. The incentive in the present study was pragmatically chosen based on promising results from an earlier pilot study and another RCT [8, 27]. In addition, and from a more practical perspective, almost all patients received social welfare, which they might lose when receiving a substantial source of extra income (e.g. ≥€30). For these reasons, we believe the amount of 30 euro is relatively adequate, but this needs to be addressed in future studies.

To conclude, our study suggests that, under certain conditions, money for medication is an ethically acceptable intervention for improving medication adherence. Issues of benefit, motivation and the size and duration of the incentive should be clarified in further research.

## Additional file

Additional file 1: ‘Appendix A. Money for medication questionnaire’. This file shows the original questionnaire that was used in this study, containing 2 open-ended questions and 19 statements which were scored on a 5-point scale (1 = strongly disagree, 5 = strongly agree). (DOCX 102 kb)

## Abbreviations

M4M: Money for Medication (intervention group)
TAU: Control group

## References

[1] Valenstein M, Ganoczy D, McCarthy JF, Myra Kim H, Lee TA, Blow FC (2006). Antipsychotic adherence over time among patients receiving treatment for schizophrenia: a retrospective review. J Clin Psychiatry.

[2] Ascher-svanum H, Ph D, Zhu B, Faries D, Lacro JP, Pharm D (2006). A prospective study of risk factors for nonadherence with antipsychotic medication in the treatment of schizophrenia. J Clin Psychiatry.

[3] Ward A, Ishak K, Proskorovsky I, Caro J (2006). Compliance with refilling prescriptions for atypical antipsychotic agents and its association with the risks for hospitalization, suicide, and death in patients with schizophrenia in Quebec and Saskatchewan: a retrospective database study. Clin Ther.

[4] Novick D, Haro JM, Suarez D, Perez V, Dittmann RW, Haddad PM (2010). Predictors and clinical consequences of non-adherence with antipsychotic medication in the outpatient treatment of schizophrenia. Psychiatry Res.

[5] Gray R, Bressington D, Ivanecka A, Hardy S, Jones M, Schulz M (2016). Is adherence therapy an effective adjunct treatment for patients with schizophrenia spectrum disorders? A systematic review and meta-analysis. BMC Psychiatry.

[6] Claassen D, Fakhoury W, Ford R, Priebe S (2007). Money for medication: financial incentives to improve medication adherence in assertive outreach. Psychiatr Bull.

[7] Staring ABP, Van der Gaag M, Mulder CL (2013). Schizophrenia and antipsychotic medication - better adherence, better outcomes?. Schizophr Res.

[8] Priebe S, Yeeles K, Bremner S, Lauber C, Eldridge S, Ashby D (2013). Effectiveness of financial incentives to improve adherence to maintenance treatment with antipsychotics : cluster randomised controlled trial. BMJ.

[9] Noordraven EL, Wierdsma AI, Blanken P, Bloemendaal AFT, Staring ABP, Mulder CL (2017). Financial incentives for improving adherence to maintenance treatment in patients with psychotic disorders (money for medication): a multicentre, open-label, randomised controlled trial. Lancet Psychiatry.

[10] Claassen D (2007). Financial incentives for antipsychotic depot medication: ethical issues. J Med Ethics.

[11] Shaw J (2007). Is it acceptable for people to be paid to adhere to medication?. BMJ.

[12] Cairns R, Maddock C, Buchanan A, David AS, Hayward P, Richardson G (2005). Prevalence and predictors of mental capacity in psychiatric inpatients. Br J Psychiatry.

[13] Promberger M, Marteau TM (2013). When do financial incentives reduce intrinsic motivation? Comparing behaviors studied in psychological and economic literatures. Health Psychol.

[14] Kendall T (2013). Paying patients with psychosis to improve adherence. Br Med J.

[15] Brown RCH. Social values and the corruption argument against financial incentives for healthy behaviour. J Med Ethics. 2017:140–4. doi:10.1136/medethics-2016-103372. 10.1136/medethics-2016-103372 PMC5339565 27738254

[16] Burton A, Marougka S, Priebe S (2010). Do nancial incentives increase treatment adherence in people with severe mental illness? A systematic review. Epidemiol Psychiatr Sci.

[17] Szmukler G (2009). Financial incentives for patients in the treatment of psychosis. J Med Ethics.

[18] Priebe S, Sinclair J, Burton A, Marougka S, Larsen J, Firn M (2010). Acceptability of offering financial incentives to achieve medication adherence in patients with severe mental illness: a focus group study. J Med Ethics.

[19] Highton-Williamson E, Barnicot K, Kareem T, Priebe S (2015). Offering financial incentives to increase adherence to antipsychotic medication: the clinician experience. J Clin Psychopharmacol.

[20] Beauchamp TL, Childress JF (2012). Principles of biomedical ethics.

[21] Bloch S, Green SA (2006). “an ethical framework for psychiatry”: comment. Br J Psychiatry.

[22] Foot P. The problem of abortion and the doctrine of the double effect. Oxford Rev. 1967:5–15. doi:10.1093/0199252866.001.0001.

[23] Burns T (2007). Is it acceptable for people to be paid to adhere to medication?. BMJ.

[24] Szmukler G, Appelbaum PS (2008). Treatment pressures, coercion and compulsion in mental health care. J Ment Health.

[25] Nelson RM, Beauchamp T, Miller V a, Reynolds W, Ittenbach RF, Luce MF. The concept of voluntary consent. Am J Bioeth 2011;11:6–16. doi:10.1080/15265161.2011.583318. 10.1080/15265161.2011.583318 21806428

[26] Dickerson FB, Tenhula WN, Green-Paden LD (2005). The token economy for schizophrenia: review of the literature and recommendations for future research. Schizophr Res.

[27] Staring ABP, Mulder CL, Priebe S (2010). Financial incentives to improve adherence to medication in five patients with schizophrenia in the Netherlands. Psychopharmacol Bull.

