# Supplementary material for: Ethical acceptability of offering financial incentives for taking antipsychotic depot medication: patients’ and clinicians’ perspectives after a 12-month randomized controlled trial
Source: BMC Psychiatry. 2017 Aug 29;17:313. doi: 10.1186/s12888-017-1485-x (PMC5576283; doi:10.1186/s12888-017-1485-x)
Supplement: Additional file 1: — ‘Appendix A. Money for medication questionnaire’. This file shows the original questionnaire that was used in this study, containing 2 open-ended questions and 19 statements which were scored on a 5-point scale (1 = strongly disagree, 5 = strongly agree). (DOCX 102 kb) [file 12888_2017_1485_MOESM1_ESM.docx]

Additional file 1: Money for Medication questionnaire

**Money for Medication**
What do you think about the M4M project? Good idea Bad idea

What do you think are the advantages of M4M? …………………………………………………………….

What do you think are the disadvantages of M4M? …………………………………………………………….

| **Strongly disagree** | **Disagree** | | **Neutral** | | **Agree** | | **Strongly agree** |
| --- | --- | --- | --- | --- | --- | --- | --- |
|  |  |  | |  | |  | |

1. To give money for depots is good

2. Giving money for depots emphasizes the things that are going well

3. Money could just be the right push to accept your depot

4. Patients will feel dependent if they receive money for their depots

5. Money for depots improves patients’ motivation to
 use depot medication

6. Money for depots will work in daily practice

7. If they receive money for their depot medication,
 patients will feel forced to accept their depots

8. Jealousy will arise if some patients receive money for their depots and others do not

9. If someone receives money for his depot, he won’t gain insight into his problems

10. Money for depots helps to get into a positive flow

| **Strongly disagree** | **Disagree** | | **Neutral** | | **Agree** | | **Strongly disagree** |
| --- | --- | --- | --- | --- | --- | --- | --- |
|  |  |  | |  | |  | |

11. Patients will accept their depots more often when they receive money

12. Money for depots is harmful to the therapeutic relationship

13. If patients no longer receive money for their depots they will stop to accept their depots

14. Money for depots will provoke patients to follow their treatment less for themselves but more for the money

15. Money for depots is beneficial for patients wellbeing

16. Giving money for depots is ethically acceptable

17. It is good to reward good behavior with money

18. It is not permissible to buy patients by giving them money to take their medication

19. If patients receive money for their depot they will accept it more often
